# Comparison of RetinaNet, SSD, and YOLO v3 for real-time pill identification

**DOI:** 10.1186/s12911-021-01691-8

**Published:** 2021-11-22

**Authors:** Lu Tan, Tianran Huangfu, Liyao Wu, Wenying Chen

**Affiliations:** Department of Pharmacy, The Third Affiliated Hospital of Southern Medical University, Guangzhou, 510000 China

**Keywords:** Convolutional neural network, RetinaNet, SSD, YOLO v3, Pill identification

## Abstract

**Background:**

The correct identification of pills is very important to ensure the safe administration of drugs to patients. Here, we use three current mainstream object detection models, namely RetinaNet, Single Shot Multi-Box Detector (SSD), and You Only Look Once v3(YOLO v3), to identify pills and compare the associated performance.

**Methods:**

In this paper, we introduce the basic principles of three object detection models. We trained each algorithm on a pill image dataset and analyzed the performance of the three models to determine the best pill recognition model. The models were then used to detect difficult samples and we compared the results.

**Results:**

The mean average precision (MAP) of RetinaNet reached 82.89%, but the frames per second (FPS) is only one third of YOLO v3, which makes it difficult to achieve real-time performance. SSD does not perform as well on the indicators of MAP and FPS. Although the MAP of YOLO v3 is slightly lower than the others (80.69%), it has a significant advantage in terms of detection speed. YOLO v3 also performed better when tasked with hard sample detection, and therefore the model is more suitable for deployment in hospital equipment.

**Conclusion:**

Our study reveals that object detection can be applied for real-time pill identification in a hospital pharmacy, and YOLO v3 exhibits an advantage in detection speed while maintaining a satisfactory MAP.

## Introduction

In China, due to medical insurance policies requirements, oral pills for inpatients are dispensed individually by inpatient pharmacies according to the prescribed dosage, and pharmacists need to disassemble the packaging of the pills for dispensing. These cases usually require unpacking the pills from their original labeled containers. However, in contrast to management systems in countries such as the United States and Japan, the China Food and Drug Administration (CFDA) does not mandate that pills have an imprint code. Therefore, as some of the solid oral dosage forms may not be clearly distinguishable from each other in terms of size, shape, or color, when the packaging is removed it may be difficult for hospital pharmacists to distinguish between pills. Similar looking pills that cannot be identified must be discarded, which results in a waste of medical resources. Solving this problem requires not only long-term knowledge and experience on the part of pharmacists, but also requires intense focus on their work. However, with China's growing and aging population, the demand for medical care is increasing [1], which places considerable pressure on the limited medical resources [2]. In most primary care hospitals, many pharmacists still dispense drugs and check them manually. Although some large hospitals have now adopted the expensive Automatic Tablet Dispensing Machine, it seems that filling errors, accidental dropping of medication into the machine, and other human errors remain unavoidable [3, 4]. 'Err is Human' [5], and even experienced pharmacists can make mistakes under the pressure of constant high intensity work. Dispensing the wrong drug will seriously compromise the safety of treatment [6, 7].

With the phenomenal development of machine learning in recent years, machine learning has been widely applied to computer vision, medical image processing, and many other fields. Some progress has been made in drug discovery [8], drug production [9] and semi-quantification [10], but very little research has been done on pill identification. As sophisticated algorithms continue to emerge, it seems likely that it will be possible to apply image processing research to pill identification. The accuracy of the model is the basic indicator that determines whether this technology can assist a pharmacist's work. In addition, the efficiency of the model is also important. For example, if the model calculation takes too long, it will not be suitable for use in a busy environment. To investigate this possibility, we trained some current mainstream object recognition algorithms, including RetinaNet, Single Shot Multi-Box Detector (SSD), and You Only Look Once (YOLO v3), on a newly created pill dataset and compared the results in terms of accuracy and detection speed, to determine the best model to assist pharmacists and other healthcare workers dispense and check drugs affordably.

## Related work

Early related research was mainly based on traditional machine learning. Lee et al. proposed a Canny edge detection and invariant moments method to extract the feature vector from pill imprint images [11]. Morimoto et al. used images captured from both-sides of tablets to identify them by matching distinctive marks [12]. Suntronsuk et al. used Otsu's thresholding with noise elimination to extract the imprint from pills as a vector, achieving precision and recall scores on the recognition of text on imprints of over 57% [13]. Neto et al. proposed a feature extractor based on shape and color in 1,000 images of 100 different classes of pills, obtaining an accuracy of over 99% using various classifiers [14]. Dhivya et al. used a support vector machine to recognize text imprinted on tablets [15].

Traditional machine learning methods achieve the detection of targets by manually designing feature learning methods, and the characteristics of the feature extraction design and classifier selection often largely determine the final detection accuracy. Hence, the corresponding characteristic parameters need to be set manually for different tablets. However, because of China's Centralized Drug Bidding and Purchase Mechanism, the same drug will be centrally tendered each year, which means that it may be supplied by different pharmaceutical companies. Therefore, due to the annual variation in the types of pills chosen, a manual approach to feature design generates a significant amount of work. This approach may lack robustness to the diversity of the pills and cannot handle large volumes. In particular, when there is no imprint code, the similar appearance of pills and the lack of the corresponding parameters can degrade recognition accuracy. Also, the traditional object detection approach uses a computationally intensive sliding window method, which makes it difficult to achieve real-time performance. Therefore, an improved solution is desirable.

Convolutional neural networks (CNN) are the most common deep learning algorithm, applying multiple convolutional layers and convolutional computation. They have efficient feature extraction capability and provide a better problem-solving method for object detection. Wong et al. used the improved AlexNet-based algorithm, which won the ILSVRC 2012 championship, and compared it with two traditional machine learning methods, k-nearest neighbors and random forests, for pill feature extraction, ultimately demonstrating the superiority of AlexNet. The results showed that the top-1 pill recognition by the AlexNet-based network performed better than those with manually designed features, reaching 95.35% [16]. However, AlexNet, as a light network with only a few layers, can only implement simple applications, and as the complexity of the task increases, it is not flexible enough to train a robust neural network for this task. Swastika et al. proposed using three LeNet or AlexNet models to extract the three main features of pills, shape, color, and imprint, and combined three CNNs into an integrated network for pill identification. The network was trained on 24,000 images of eight types of pill, achieving a recognition accuracy of up to 99.16% [17]. Ou et al. proposed a drug pill detection system similar to a two-stage target detection algorithm based on ResNet for localization detection and Xception for classification. The training set included 131 categories and a total of 1,680 images for training. The top-1 accuracy rate for the trained network was up to 79.4% [18]. Based on these studies, deep learning has gradually replaced manual design extraction in pill feature extraction, and deep learning algorithms, such as LeNet, AlexNet, and ResNet, are able to address the problem of pill image classification. The CNNs used for target detection, such as Retinanet, SSD, and YOLO architectures, incorporate the structure of the above-mentioned CNNs used for image classification, and can accomplish both image classification and target localization, but they have not been applied to pill identification. In addition, in practical applications, especially in places with high workloads such as pharmacies, there is a need to consider accuracy while also focusing on preforming the task in real-time. To the best of our knowledge, existing studies do not take real-time performance into account.

### Object recognition technology based on deep learning

Current approaches using deep learning methods for target classification and regression can be divided into two categories. One is the two-stage algorithm represented by architectures such as R-CNN, Fast R-CNN, and Faster R-CNN. This type of algorithm is usually carried out in two steps. The first one is to use a selective search or Region Proposal Net (RPN) to generate possible target regions, and then complete classification and regression on Region Proposal. This method has high accuracy but also limits the detection speed. Another algorithm is the one-stage algorithm, which is represented by RetinaNet, SDD, or YOLO. The one-stage algorithms use a single network to directly predict object bounding boxes and class probability scores from images. The detection speed is improved by avoiding the use of RPNs. However, the accuracy of the one-stage algorithm for small target detection is not as good as the two-stage algorithm. The detection accuracy and detection speed of the model directly affect the feasibility of pill recognition.

**Fig. 1:**
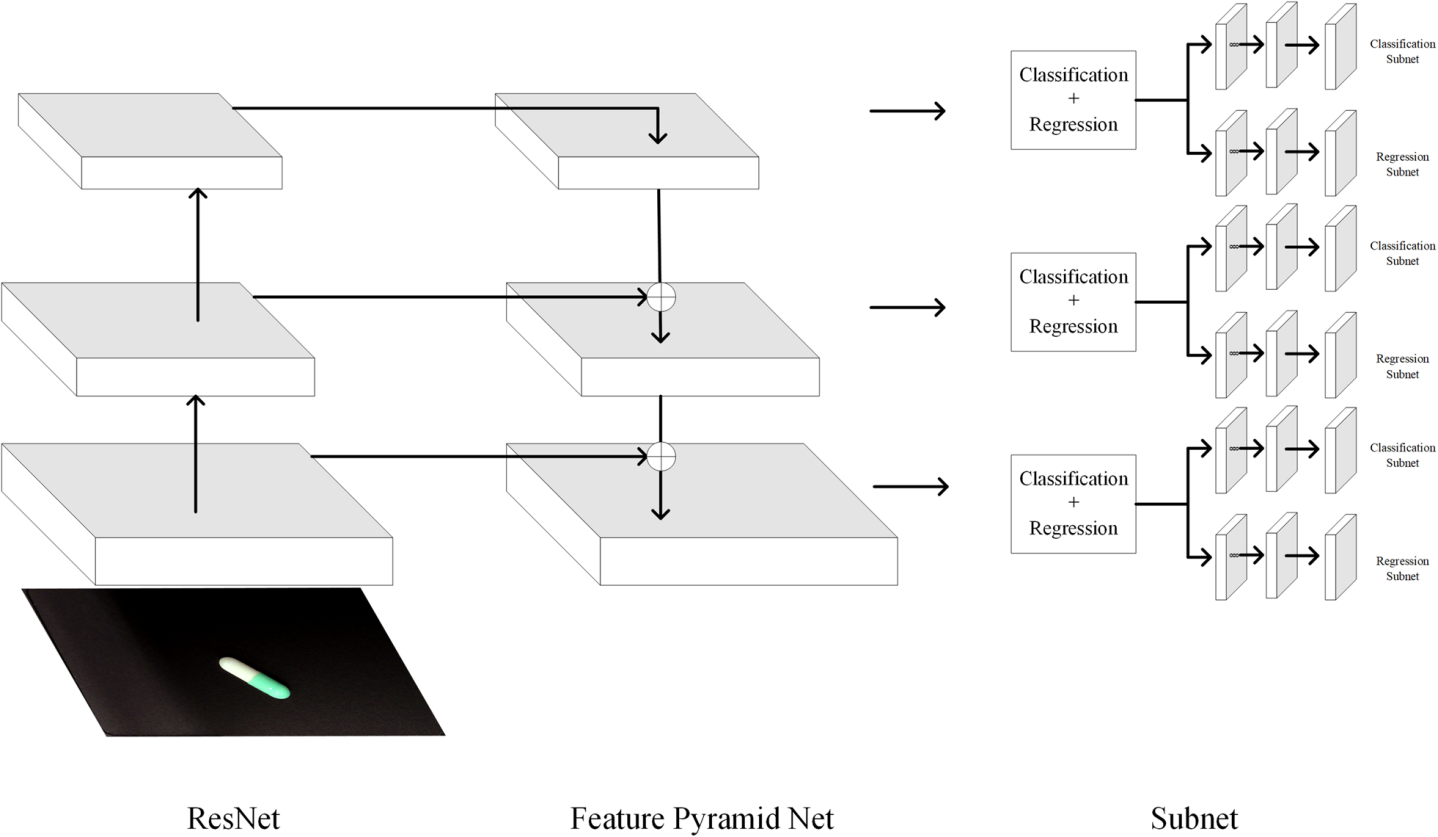
RetinaNet structure

RetinaNet is one of the representative one-stage algorithms and the structure is shown in Fig. 1. The backbone uses ResNet and Feature Pyramid Net (FPN) structures. Based on the FPN structure, a top-down path and horizontal connection are added. Each level of the FPN is connected to the fully convolutional networks, which include two independent subnets that are used for classification and regression. The main innovation of RetinaNet is the addition of Focal Loss to the Classification Subnet. Since the imbalance of the number of positive and negative samples in the target detection of the one-stage algorithm will affect the training loss, Focal Loss assigns different weights to hard samples, which effectively solves the class imbalance problem in the target detection model. A study [19] in 2017 showed that RetinaNet could achieve detection speeds similar to some one-stage algorithms, and the detection accuracy exceeded many two-stage algorithms at that time.

**Fig. 2:**
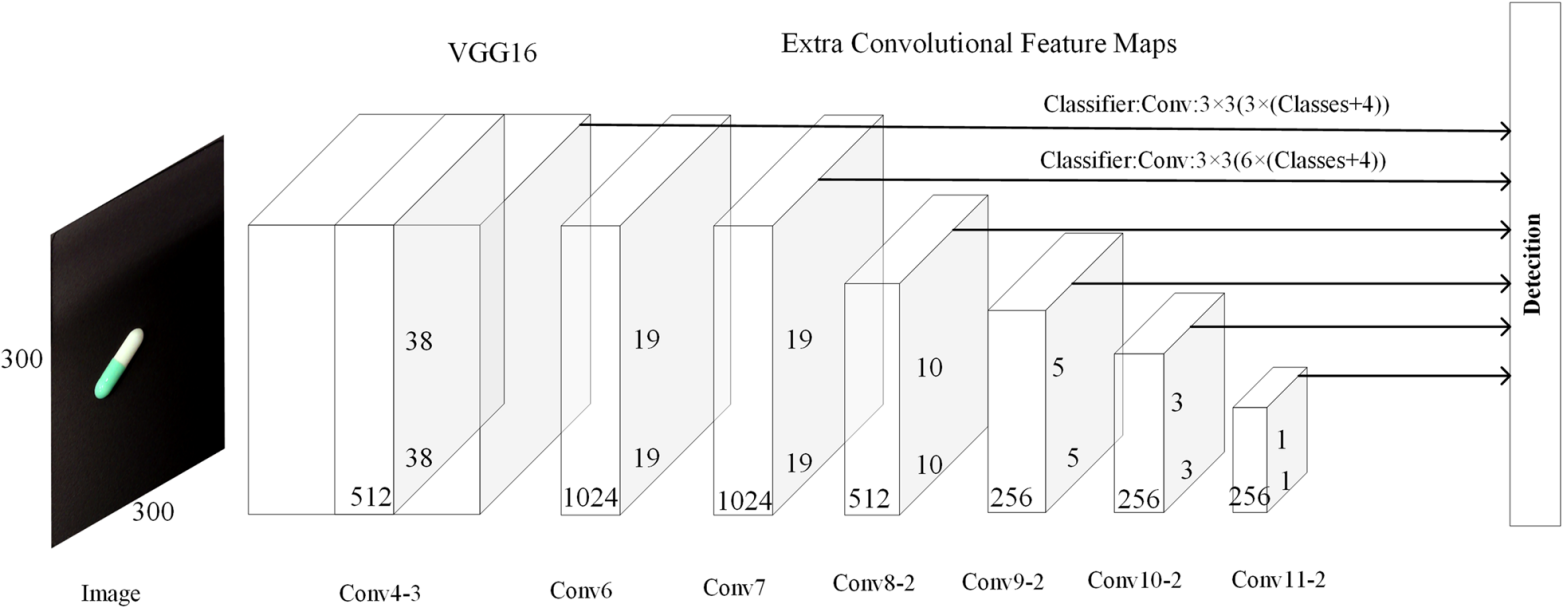
SSD network structure

SSD [20] was proposed by Wei Liu et al. and draws on the anchor mechanism of Faster R-CNN and the end-to-end one-step structure of the YOLO algorithm in which object classification and location regression are performed directly in the convolution stage. The main network of the SSD algorithm is shown in Fig. 2. SSD uses the VGG-16 network as a backbone and modifies it by replacing the last two fully connected layers with convolutional layers while also adding another four convolutional layers later to finally form the feature extraction network as Conv4_3, Conv7, Conv8_2, Conv9_2, Conv10_2, and Conv11_2, whose sizes are (38, 38), (19, 19), (10, 10), (5, 5), (3, 3), and (1, 1), respectively. SSD is trained to obtain a set of fixed-sized bounding boxes and the class prediction scores of the targets in the bounding boxes. Then, redundant bounding boxes are filtered out and the final detection results are generated by the non-maximum suppression (NMS) algorithm, which has good results both in terms of speed and accuracy of detection.

**Fig. 3:**
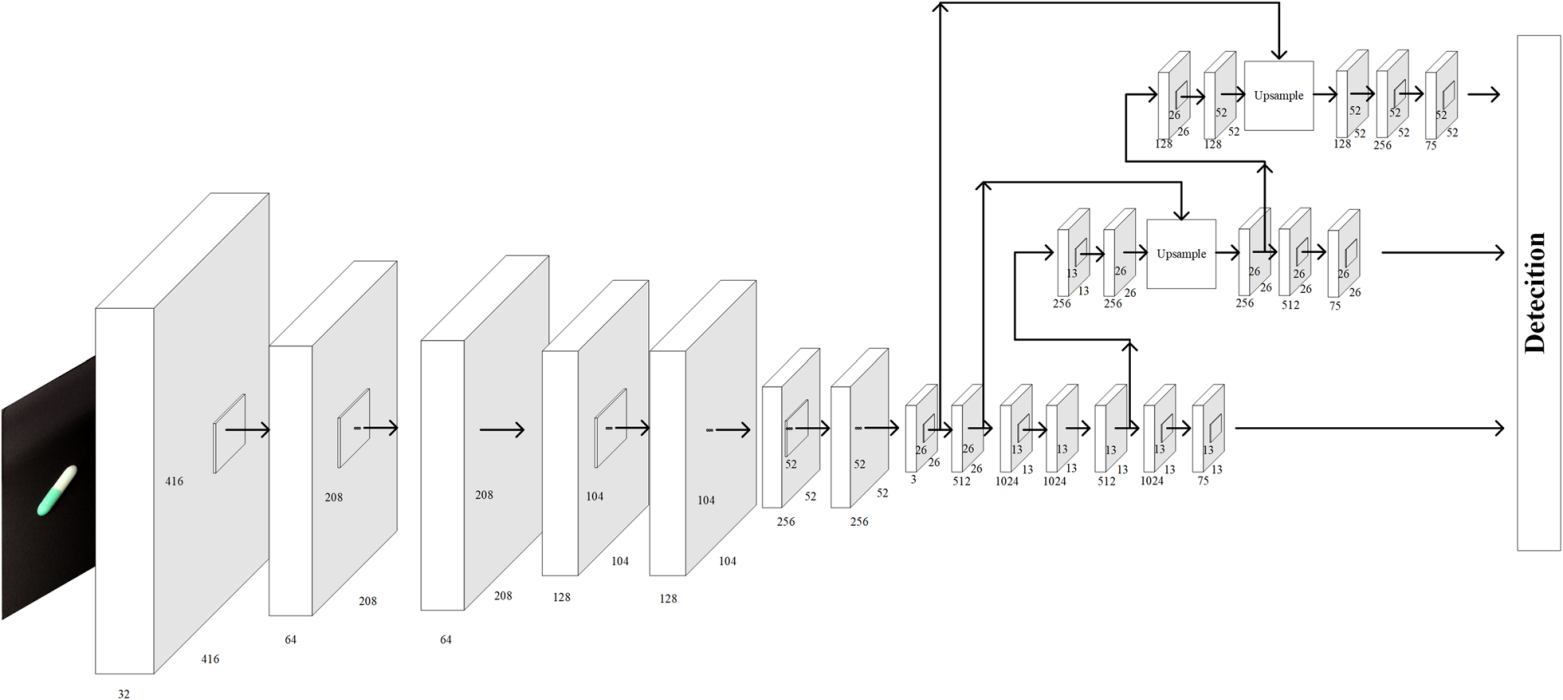
YOLO v3 network structure

YOLO [21] proposes a new idea for target detection by transforming the task into a regression problem. The whole framework only needs to use a relatively simple CNN structure to directly complete the regression of target detection to predict the position of the bounding box and the class of the candidate box. The YOLO v3 [22] backbone network structure does not have the pooling and fully connected layers, as shown in Fig. 3, and the convolutional transformation of the image is achieved by changing the step size of the convolutional core. YOLO v3 uses Darknet-53 as the network skeleton, which makes the network structure deeper and better at extracting features, as demonstrated by its improved accuracy compared with YOLO v1 and YOLO v2. Darknet-53 makes extensive use of the ResNet residual structure, which can avoid the vanishing gradient problem even when the network structure is deep.

## Methods

### Dataset preparation

The training of deep learning models typically requires many data samples to obtain reliable parameters and models. In 2016, the U.S. National Library of Medicine published an algorithm challenge competition on pill recognition, and publicly released the pill image dataset [23]. However, considering our particular situation in which there are some kinds of pills without an imprint code, this dataset was not considered suitable. Therefore, we decided to create our own dataset for use in this experiment.

**Fig. 4:**
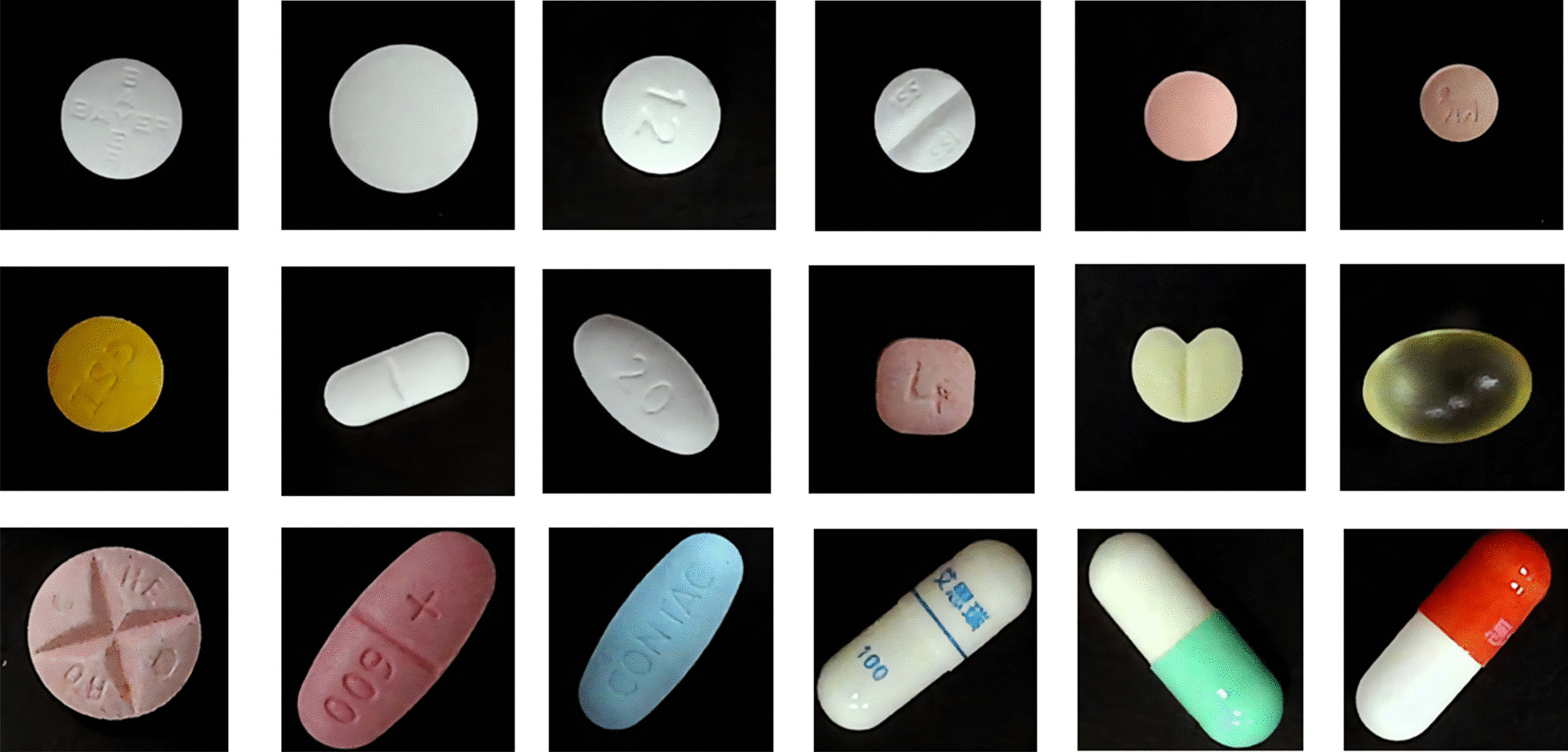
Example images of solid oral dosage forms

The appearance of our existing oral solid dosage forms was analyzed by observation, and images were taken using a high-speed photographic apparatus connected to a computer. The pills were placed at a random location on the board. Since the height of the high-speed photographic apparatus is fixed, the distance of each pill shot is also relatively constant. Each pill shot includes both front and back images, for a total of 5,131 images. The statistics of the dosage form, printing, shape, color, and manufacturer of the pills. There was a total of 261 varieties of oral solid drugs commonly used in inpatient pharmacies, including 70 capsules and 191 tablets, as shown in Table 1. We observed that some pills have a special code, manufacturer's trademark image, and that several of them were printed at the same time after removing the packaging, which aids in identification. However, there are still some tablets that are hard to distinguish after removing the outer packaging. Representative images of the tablets are shown in Fig. 4.

**Table 1:** Appearance of pills

Dosage form	Printing	Non-round shape	Non-round appearance	Total number of pill varieties
Naked tablet	2	0	7	21
Sugar coated tablet	1	0	8	14
Film-coated tablet	111	66	66	156
Capsule	34	–	55	61
Soft capsule	1	–	8	9
Total	149	66	144	261

### Object image annotation

**Fig. 5:**
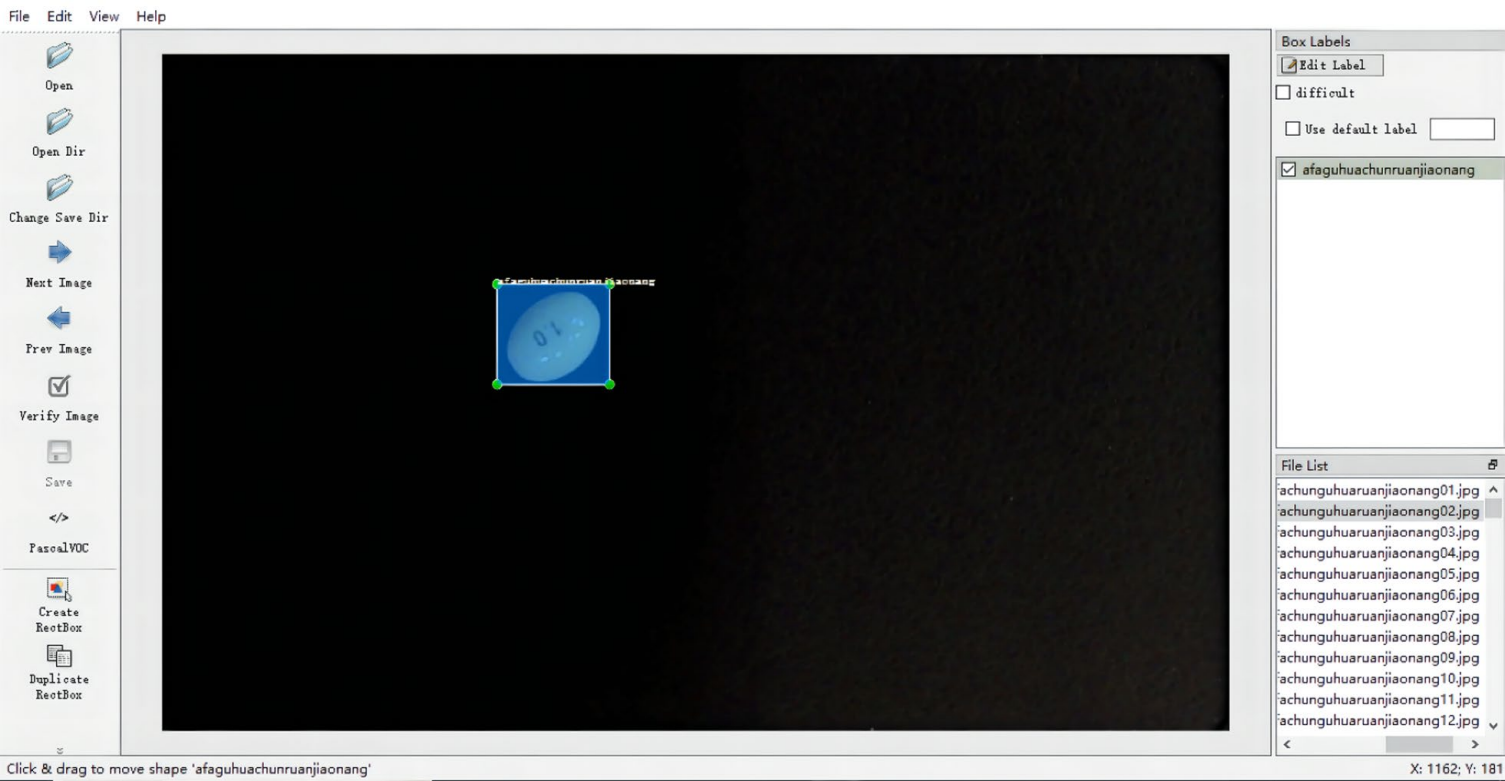
LabelImg tool for image labeling

Since the object recognition method used in this experiment is a type of supervised learning, it is necessary to obtain the labeling information of the pill to be detected in the image; this includes the pill category information and the pill border location information. LabelImg is written in Python. Since the labeling format of LabelImg is consistent with PASCAL VOC and has a good graphical interactive interface with a rich array of shortcut keys, it was used to improve the labeling efficiency in our experiment. The image annotation process is shown in Fig. 5. After labeling the tablets with LabelImg, the information of each image is saved in an "xml" file with the same name. The xml file contains all the information needed for training the network, including the class of the object and the location of the object in the image. After the image annotation work was completed, the image dataset was enlarged to 51,310 images by means of horizontal flip enhancement, vertical flip enhancement, mirror symmetry enhancement, affine change, rotation, Gaussian noise addition contrast change, and scale transformation.

### Training models

**Fig. 6:**
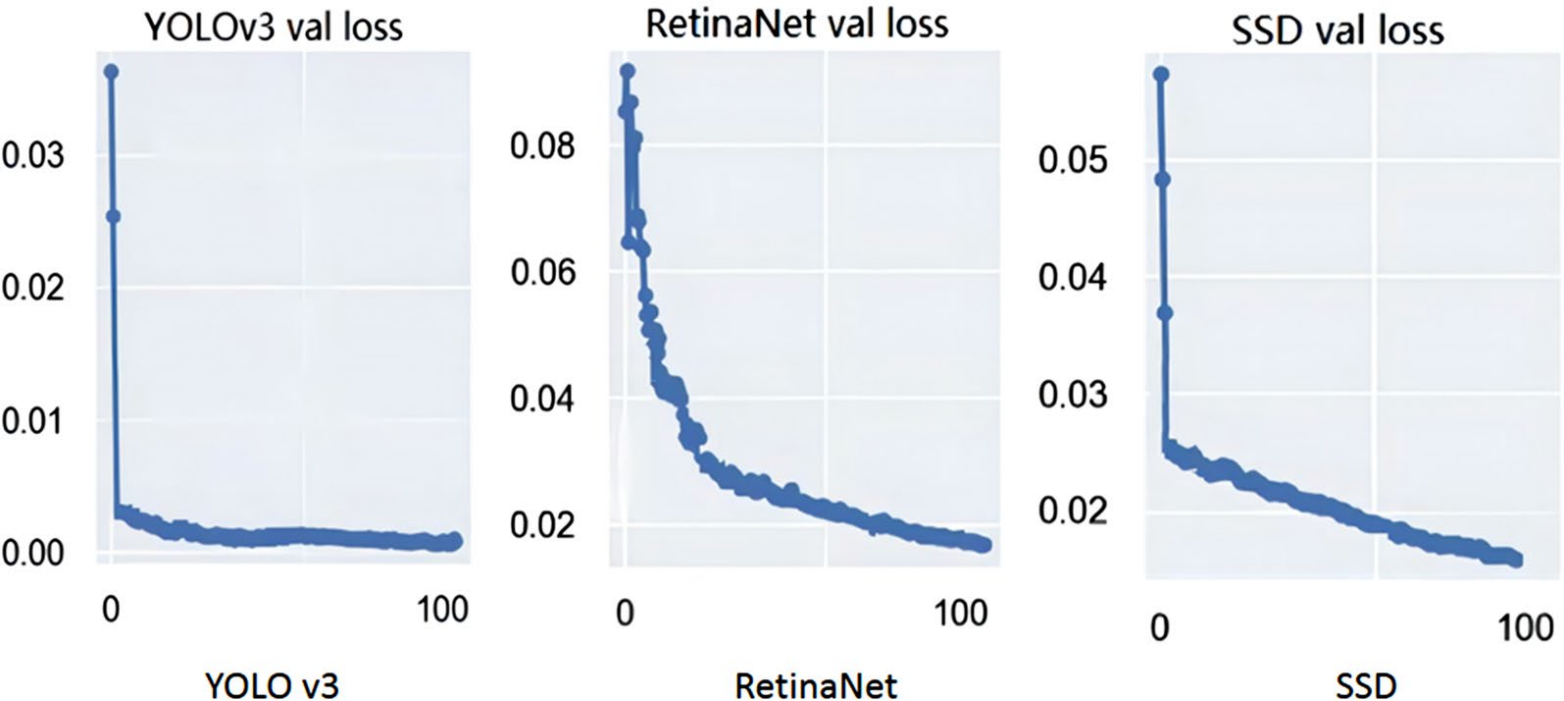
Graph of Loss function

The experimental platform configuration for this paper is the following: OS, Win10; GPU, NVIDIA GeForce GTX 1080Ti; CPU, Intel(R) Core(TM) i7-7700 K CPU @ 4.20 GHz. The experimental platform was built based on the Python programming language and the PyTorch framework.All three models were trained on this configuration. The specific parameters are shown in Table 2. The models were all pre-trained on the Ms. COCO dataset, and then transferred to the pills dataset for training. After the training parameters were set, the dataset was divided according to the ratio of 6: 2: 2 for the training set, validation set and testing set, respectively.The training set was used to train the model and the validation set was used to check the state of the model during the training process to assess whether the model was over-fitting. After the training was completed, the test set was used to evaluate the generalization ability of the model. The value of the loss function is shown in the Fig. 6. When the training starts, the descent gradient increases rapidly, but then the change in Loss value gradually slows down, and finally stabilizes.

**Table 2:** Parameter configuration

Parameter	Value
Batch	64
Sub-divisions	16
Learning rate	0.001
Momentum	0.9
Decay	0.0001

### Evaluation indicators

To compare the results of the three deep learning-based models for pill recognition, we applied a range of standard metrics commonly used to evaluate these models. There are four possible outcomes based on the output categories of the test samples compared with the categories of the true labels, as follows: true positives (TP), false positives (FP), false negatives (FN), and true negatives (TN). If the target type is detected correctly, the center coordinates of the detection frame and the dimensions of the detection frame are within tolerable limits, then the detection result is recorded as TP. FP refers to a target category recognition error or the detection frame is not within the preset threshold. The predicted result of a target that is not detected is recorded as FN. As we did not predict the absence of a pill, the category of TN was not used.

The observed counts are combined into standard metrics including recall, precision, F1 score (F1), mean average precision (MAP), and frames per second (FPS). In the process of target detection, precision is the ratio of correctly detected targets to the number of all detected targets; recall is the ratio of the number of correctly detected targets to all targets in the sample set. The definition of precision and recall are shown in Formulas 1 and 2, respectively:

1 $$Precision = \frac{TP}{{TP + FP}}$$

2 $${\text{Re}} call = \frac{TP}{{TP + FN}}$$

F1 is the weighted harmonic average of precision and recall. Since the amount of data for each pill is not the same, the F1 score is used to evaluate performance. The F1 score can be calculated from the precision and recall rates, as defined in Formula 3:

3 $$F_{1} = \frac{2PR}{{P + R}}$$

Average precision (AP) is the precision across all elements of a category of pills, as defined in Formula 4:

4 $$AP = \int\limits_{0}^{1} {p(r)dr}$$

MAP is numerically equal to the average value of the AP sum across all categories, and this value is used to evaluate the overall performance of the model. The definition is shown in Formula 5:

5 $${\text{MAP}} = \frac{1}{n}\sum\limits_{{{\text{i}} = 1}}^{n} {AP_{i} }$$

FPS is a common indicator for evaluating the speed of model detection. This refers to the number of images that can be processed per second. In general, FPS over 30 is considered to have achieved real-time detection.

## Results and discussion

### Comparison of algorithm detection results

**Fig. 7:**
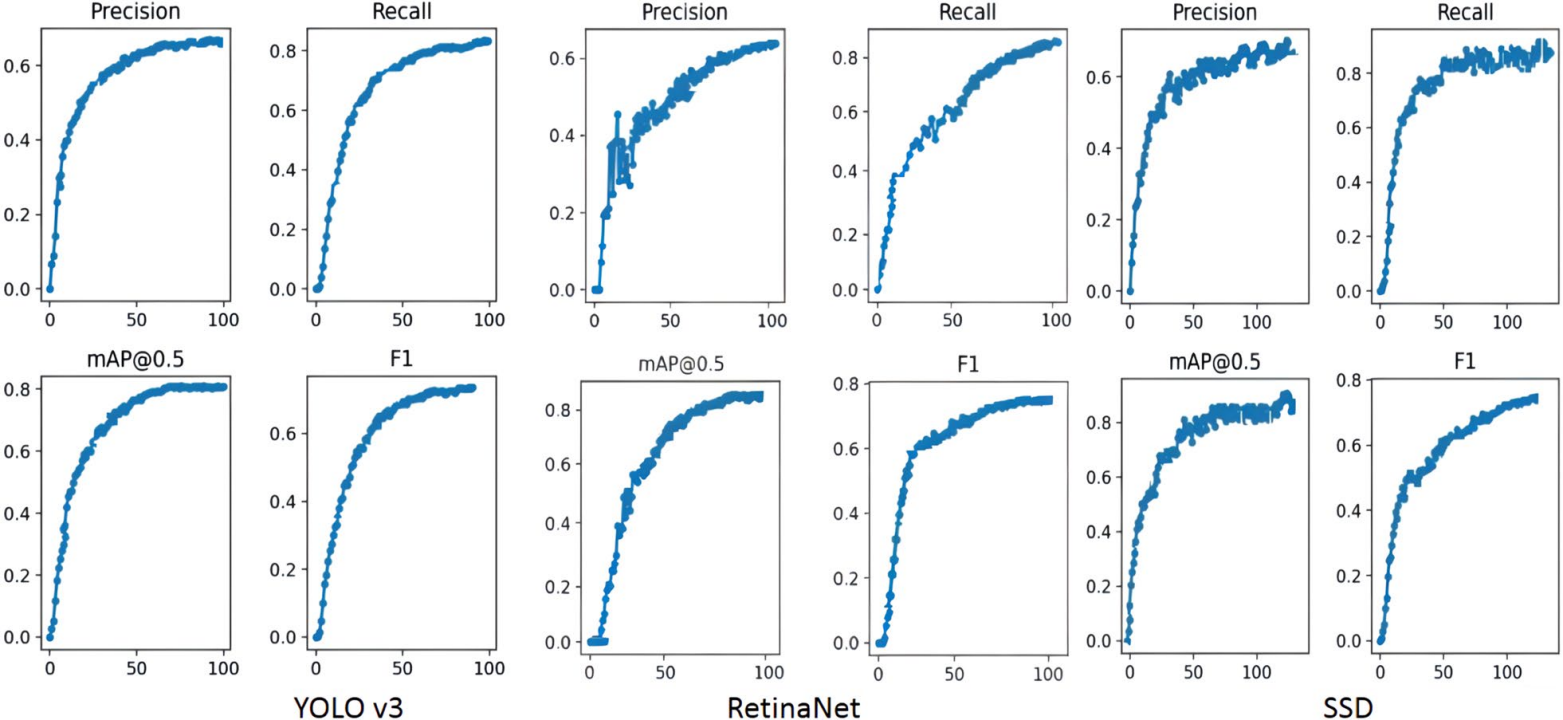
Graph of model performance measures

**Fig. 8:**
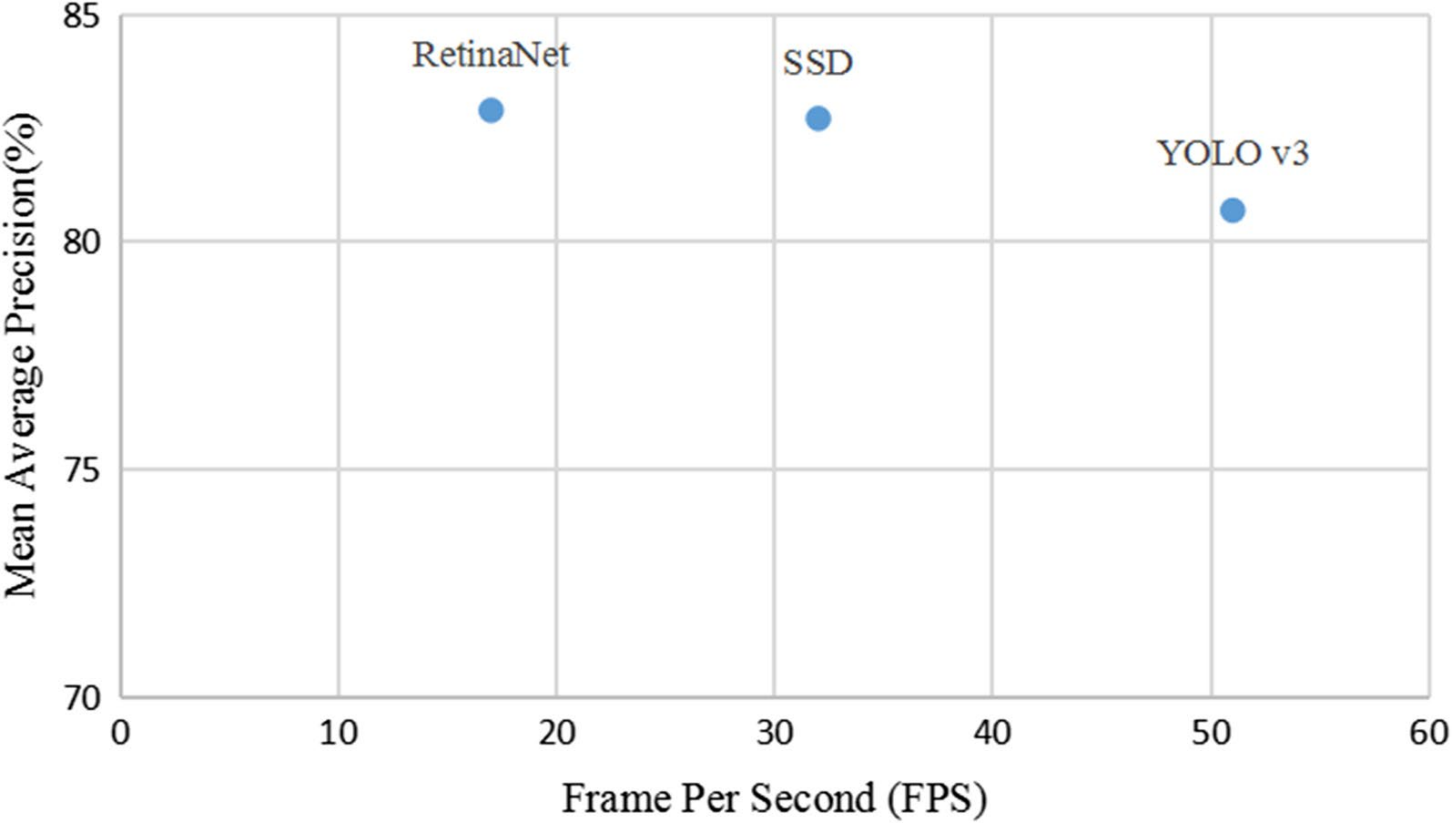
Performance of deep learning model

After training, the different algorithms were used for pill identification on the test set; the results are shown in Fig. 7 and Table 3. Compared with YOLO v3 and SSD, RetinaNet has a higher MAP by 2.20% and 0.18%, respectively. However, YOLO v3 can predict multiple bounding boxes and their categories simultaneously, and the detection speed is faster than that of the other network model structures. As shown in Fig. 8, YOLO v3 detects 51 images per second, and SSD detects 32 images per second. The detection speed of these two algorithms exceeds 30 FPS, which is much faster than RetinaNet. If detection efficiency is considered, YOLO v3 performs best among the three models, while RetinaNet does not meet the real-time requirements, which limits its potential applications. Based on the analysis of the above experimental results, RetinaNet is more suitable if the higher MAP of pill recognition is required, but YOLO v3 may be more suitable for use when the priority is real-time performance and it is feasible to accept a slightly lower MAP. Therefore, we believe that YOLO v3 has the potential to be applied to assist pharmacists to identify pills in a hospital dispensary environment. The precision of the three models is lower than the recall score because after removing the packaging of the tablets, part of the identifiable information is removed. Faced with many pills of similar colors or shapes, it is difficult to distinguish them from each other even for human experts. This task is also challenging for convolutional neural networks. Due to the level of difficulty, the model sometimes identifies pills incorrectly, which leads to an increase in the FP score. From Formulas 1 and 2, the increase in FP will lead to a decrease in precision, while recall is not affected by the FP rate.

**Table 3:** Evaluation of deep learning models

Algorithm	Precision (%)	Recall (%)	F1 (%)	MAP (%)
RetinaNet	64.98	83.86	73.26	82.89
SSD	63.69	88.89	74.21	82.71
YOLO v3	69.65	80.67	74.77	80.69

### Hard sample detection comparison

**Fig. 9:**
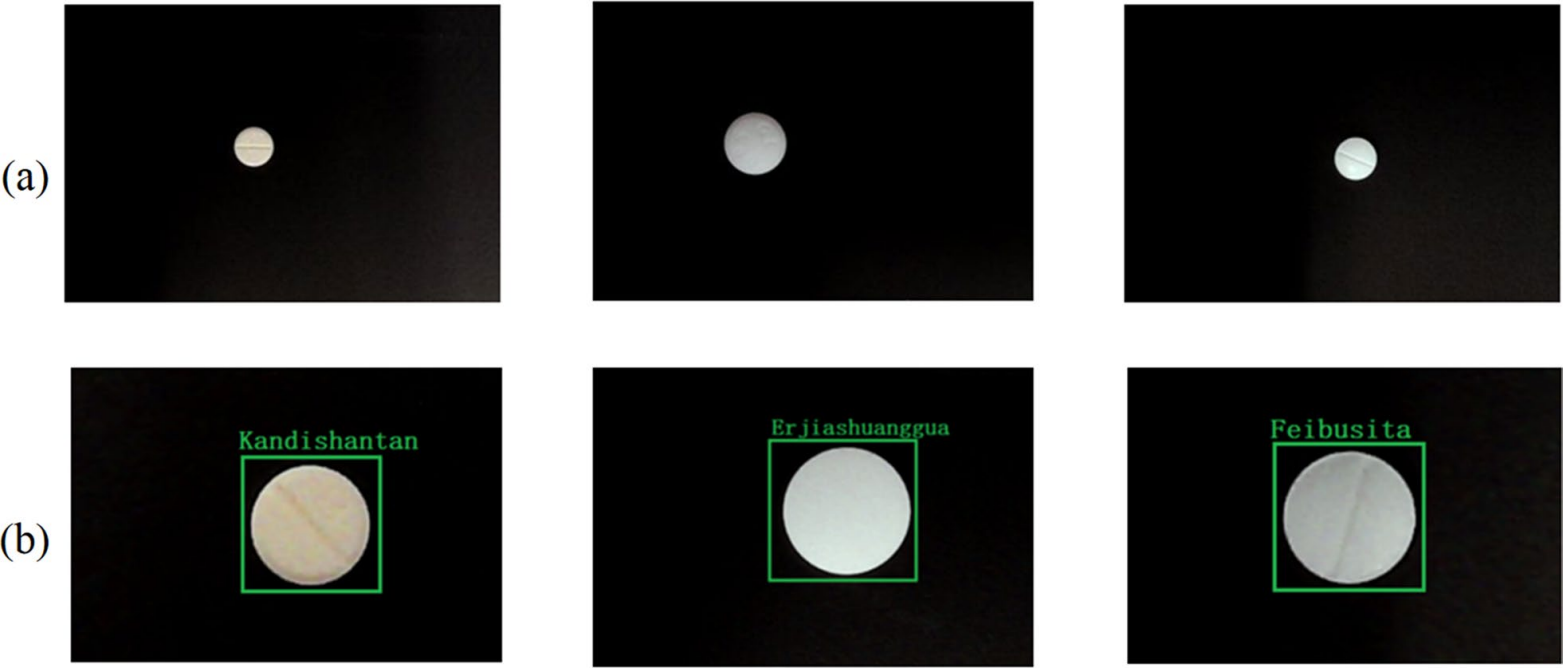
Actual detection effect of the model **a** hard samples, **b** YOLO v3 detection results

To more effectively reflect the effect of the model in identifying tablets with similar colors and shapes, we selected some tablets that are particularly hard to identify. As shown in Fig. 9a, since the tablets are small and have no obvious printed codes, they are visually more difficult to distinguish, and Fig. 9b showed the detection effect of the YOLO v3. Results from the hard to identify group are shown in Table 4. The three algorithms exhibit little difference in the MAP, but YOLO v3 has obvious advantages in FPS and model size. Features that cannot be distinguished visually can be learned through training (back-propagation), using the convolution kernel in the CNN. The features learned by the network can then be used as the basis for correct judgment of the type of pills, which greatly accelerates manual dispensing and checking. In the pharmacy, we can set the confidence threshold to assist the pharmacist in judging the medicine. When the probability (confidence) that the network judges that the current pill belongs to a certain category is lower than our set value, we interpret that the network model is having difficulty judging the current pill, and at this time, pharmacists can participate manually to ensure correctness.

**Table 4:** Indicators of models in identifying hard samples

Algorithm	MAP (%)	FPS	Model size
RetinaNet	79.61	22	157M
SSD	79.03	41	149M
YOLO v3	79.02	69	89M

## Conclusion

We collected pill images and used LabelImg to create a standard PASCAL VOC format image database. Three currently dominant object detection methods, RetinaNet, SSD, and YOLO v3 were trained using the pill dataset. The loss function of YOLO v3 converges faster, indicating that the training time of the YOLO v3 model is shorter than that of the other two models. Hence, it can better deal with the impact of retraining the model due to frequent changes of pills in pharmacies. By comparing the evaluation indicators, each of the three models has its own advantages and disadvantages. RetinaNet has a high MAP (82.89%), but the detection speed (FPS: 17) is not fast enough for real-time application. SSD is intermediate in performance, with scores between the other two networks on both speed (FPS: 32) and MAP (82.71%). Although YOLO v3 does not have the highest MAP (80.69%), it can greatly improve the detection speed and achieve real-time performance (FPS: 51). In busy hospital pharmacies, pill identification requires not only a high enough MAP, but also a fast detection speed. YOLO v3 may be the best compromise. This method can quickly help pharmacists identify drugs, reduce the probability of dispensing the wrong drug, and therefore can help improve patient safety. On the basis of model size, the YOLO v3 network can meet the requirements of the low-performance platforms and provides fast detection speeds. Therefore, it has broad development prospects and practical application value.

There are some shortcomings in our study, such as limitations in the experimental dataset, as we have only collected images of split pills from one hospital. A larger dataset would make the results more robust. Another important factor is that some different types of oral solid dosage forms currently in clinical use have a very similar appearance, which will reduce the MAP of model recognition. In future work, we will build larger datasets and keep testing new algorithms to further optimize the model and improve both the MAP and speed of detection.

## Data Availability

The dataset used in the current study is available from the corresponding author upon request.

## Abbreviations

AP: Average Precision
CFDA: China Food and Drug Administration
CNN: Convolutional Neural Network
F1: F1 score
FN: False Negatives
FP: False Positives
FPN: Feature Pyramid Net
FPS: Frames Per Second
MAP: Mean Average Precision
RPN: Region Proposal Net
TN: True Negatives
TP: True Positives
SSD: Single Shot Multi-Box Detector
YOLO v3: You Only Look Once v3
